# Identifying and Treating Those at Risk: Disparities in Rapid Relapse Among TNBC Patients in the National Cancer Database

**DOI:** 10.1245/s10434-024-15507-2

**Published:** 2024-06-13

**Authors:** Saurabh Rahurkar, Pallavi Jonnalagadda, Daniel Stover, Barbara Andersen, Demond Handley, Mohamed I. Elsaid, J. C. Chen, Samilia Obeng-Gyasi

**Affiliations:** 1 Department of Biomedical Informatics, College of Medicine, The Ohio State University, Columbus, OH USA; 2 The Center for the Advancement of Team Science, Analytics, and Systems Thinking in Health Services and Implementation Science Research, The Ohio State University, Columbus, OH USA; 3 Department of Internal Medicine, The Ohio State University, Columbus, OH USA; 4 Department of Psychology, The Ohio State University, Columbus, OH USA; 5 Division of Surgical Oncology, Department of Surgery, The Ohio State University, Columbus, OH USA

**Keywords:** Triple negative breast cancer, Social determinants of health, Health disparities

## Abstract

**Purpose:**

This study was designed to characterize features of rapid relapse TNBC (rrTNBC), an aggressive, poor prognosis breast cancer subset using the National Cancer Database (NCDB).

**Methods:**

Patients diagnosed with TNBC between 2010 and 2019 within NCDB were included in analyses. rrTNBC was defined as all-cause mortality ≤24 months from diagnosis. Patient demographic, tumor, and treatment association with rrTNBC were evaluated in univariate, bivariate analyses, and multiple logistic regression models. Two-part models are used to compare receipt of treatment (i.e., receipt of both chemotherapy and breast surgery) versus not in its relationship with rrTNBC.

**Results:**

Overall, 14.5% of patients were categorized as rrTNBC. Age older than 75 years (−41.3%), Black race (−1.4%), Medicare (−2.6%), and Charlson-Deyo score ≥2 (−4.9%) were associated with a lower probability of receiving both chemotherapy and breast surgery. Not receiving both treatments (vs. receiving both chemotherapy and breast surgery) was associated with a two-to-three-fold higher probability of rrTNBC among patients aged older than 75 years (16.6% vs. 6%), having Medicare (3.6% vs. 1.6%), and Charlson-Deyo score ≥2 (16.6% vs. 5.9%).

**Conclusions:**

Age, insurance, and comorbidity were related to a lower likelihood of treatment; yet receiving treatment reduced the risk of rrTNBC threefold for each. These findings might be valuable to inform clinical care delivery, as well as future research that examines treatment protocols among diverse patients.

Triple-negative breast cancer (TNBC) is characterized by the absence of estrogen (ER), progesterone (PR), and human epidermal growth factor (HER 2) receptors on tumor cells.^1^ Despite significant improvements in breast cancer diagnosis and treatment, patients with TNBC continue to have higher mortality rates compared with patients with other molecular subtypes (e.g., hormone receptor-positive breast cancer).^2^ Specifically, patients with TNBC have 5-year survival rates of 77% compared with 93% in other subtypes.^3^ The National Cancer Institute’s Surveillance, Epidemiology and End Results (SEER) program estimates that approximately 10% of breast cancers diagnosed between 2015–2019 were TNBC.^4^ Tumor genomics, treatment types, and social determinants of health (SDH) have been implicated in the disease process and subsequent outcomes (e.g., mortality).^5–8^

A genomic study by Zhang et al. identified a subset of patients with nonmetastatic TNBC with an aggressive clinical course characterized by treatment resistance, recurrence, and mortality within 24 months of diagnosis, called rapid relapse (rrTNBC).^6^ Furthermore, patients experiencing rrTNBC had lower immune signatures, i.e., lower antitumor immune response, relative to those without rapid relapse.^6^ Additional research suggests patients with rrTNBC are more likely to be on Medicaid or uninsured, single, non-Hispanic Black, and present at more advanced stages of disease.^6,8,9^ Furthermore, they were less likely to receive chemotherapy or surgical management.^8^ Collectively, these studies suggest that the etiology of rapid relapse is a complex interplay between tumor genomics, SDH, receipt of treatment, and response.

Extant research on rapid relapse has largely focused on data from the SEER program and the National Comprehensive Cancer Network (NCCN).^8,9^ This approach limits understanding of rrTNBC, because SEER encompasses less than half the U.S. population and lacks information on patient comorbidities.^10^ While comorbidity information is available in the NCCN, previous rrTNBC research on NCCN was based on data from only ten academic medical centers, limiting its generalizability.^9^ Because this research used older data, the impact of contemporary TNBC treatment changes (e.g., immunotherapy or other new systemic regimens) on clinical outcomes is not fully captured.^9^ In contrast, the National Cancer Database (NCDB) captures 72% of newly diagnosed cancer patients in the United States and includes patient comorbidities, detailed information on treatment receipt, and staging.^11^ Therefore, the purpose of this study was to explore risk factors for the development of rrTNBC. In this study, we will 1) describe the sociodemographic, clinical, and treatment characteristics of patients with TNBC experiencing rapid relapse versus no rapid relapse in the NCDB, 2) characterize patients with TNBC in the NCDB who receive both chemotherapy and surgical treatment, and 3) define the characteristics of TNBC patients experiencing rapid relapse based on whether they received both chemotherapy and surgical treatment. Insights from our study might help healthcare providers and policymakers identify populations of TNBC that are at greater risk of rapid relapse and address potential disparities in access to care.

## Methods

Our study focused on patients diagnosed with TNBC between 2010 and 2019 with American Joint Commission on Cancer (AJCC) clinical stage IB to IIIC, who had at least 24 months of follow-up in the NCDB. Stage IA and IV, as well as inflammatory cancers, were excluded from our analysis, because they are different in terms of treatment guidelines and prognosis. We also excluded patients of TNBC who were alive but did not have at least 24 months of follow-up for any reason.

### National *Cancer* Database

The National Cancer Database (NCDB) is a joint program of the American College of Surgeons, Commission on Cancer (CoC), and the American Cancer Society. This analysis is based on the 2019 Participant User File comprising patients diagnosed with TNBC between 2010 and 2019. Patients of TNBC were identified based on absence of ER, PR, and HER2.

Sociodemographic variables included age, race, Hispanic ethnicity, insurance status, and location of residence. We also included SDH available in the NDCB: quartiles of each of the straight-line distance between the patient’s residence and treatment facility where the cancer was diagnosed, percentage of the population with no high-school education at the area level, and median income at area level.^12^ We used recoded age in years (<45, 45–55, 55–65, 65–75, 75 and older), and race (American Indian or Alaska Native, Asian, Black, Hawaiian, and other Pacific Islander, other, White) as categorical variables. Race and ethnicity in this study are social constructs and not reflective of genetic ancestry.^13^

Clinical variables included the AJCC clinical stage classification, tumor histology (recoded as ductal, lobular, and other types of tumors), and tumor differentiation. Comorbid conditions are represented using the Charlson-Deyo Comorbidity Index (CCI).^12^ Treatment data included breast surgery, axillary surgery, chemotherapy, and radiation therapy. We categorized breast surgery types as no surgery, lumpectomy, and mastectomy. Axillary surgery was defined as no axillary surgery, sentinel lymph node biopsy (SLNB), and axillary lymph node dissection (ALND), which was defined as the removal of >10 lymph nodes.^14^ We also included the type of treatment facility.^12^

Two outcome variables—rapid relapse and treatment—were studied. Consistent with definitions used in large TNBC cohort studies,^9,15–18^ rapid relapse was operationalized as all-cause mortality at or less than 24 months from TNBC diagnosis, which represents relapses of TNBC as well as deaths from TNBC. We operationalized treatment as a binary variable (1: patients received chemotherapy AND breast surgery, 0: patients received chemotherapy OR breast surgery OR neither)

### Ethics Approval

The Ohio State University Office of Responsible Research Practices deemed this study institutional review board exempt.

#### Statistical Analysis

We first examined all variables and their distributions using univariate analyses. Next, we examined the relationships between our independent variables and each of our dependent variables in bivariate analyses using chi-square test. In multivariable analysis, we modeled all-cause mortality (rapid relapse) and independent variables (sociodemographic, area level SDH, clinical, treatment, and type of facility) using logistic regression. Additionally, a two-part logistic regression model was used to examine how receipt of treatment covaried with all-cause mortality (rapid relapse). In the first part of this model, we used logistic regression to analyze the relationship between receipt of treatment and select independent variables (sociodemographic variables, area level SDH variables, clinical variables, radiation therapy, and type of treatment facility). In the second part, we performed separate logistic regressions to examine all-cause mortality (rapid relapse) among those who received treatment and those who did not receive treatment. Covariate selection for all models was informed by prior literature.^8,9^ We used robust standard errors to account for the clustering of outcomes within each facility and to address heteroscedasticity resulting from this. Results are presented as average marginal effects (absolute difference from baseline, %) with 95% confidence intervals for more intuitive interpretation.^19,20^ Furthermore, marginal effects also allow quantification of the incremental risk for the dependent variable associated with each independent variable.^21^ All analyses were performed by using Stata/SE 17.0.

## Results

**Fig. 1:**
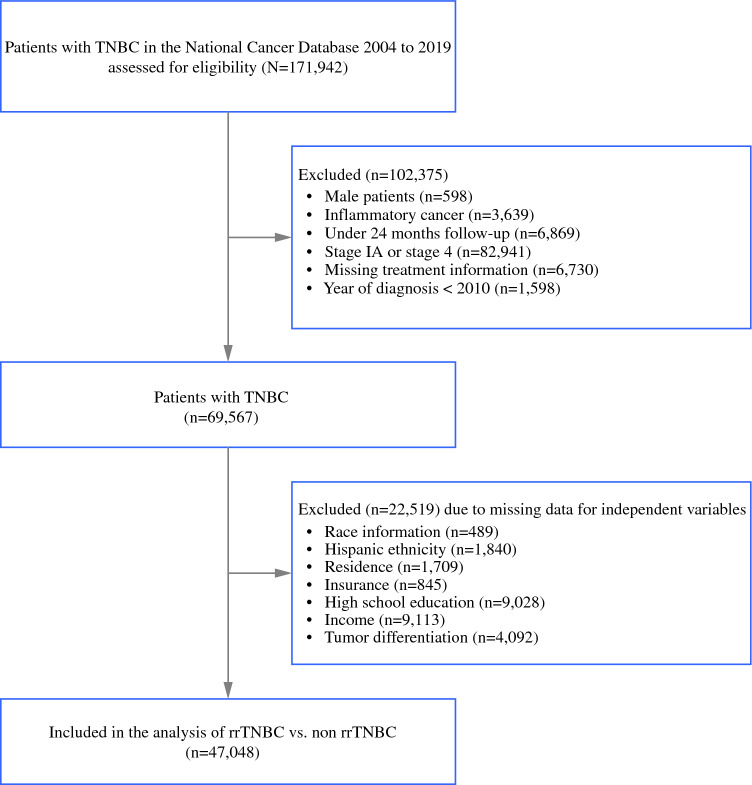
Study population from NDCB

Of the 171,942 patients with TNBC in the NCDB, a total of 47,048 patients with TNBC met our inclusion criteria and did not have missing data (Fig. 1). Most patients with TNBC (Table 1) were between 45–64 years old (56.2%), identified as White (71.5%), and had private insurance or managed care (51.8%). Almost half of the patients lived in neighborhoods where more than 10.9% of the population had less than high-school degrees (49.5%), and median incomes were <$50,353 (41.9%). Most patients with TNBC had low CCI scores of 0 (80.6%), had stage I–II disease (81.5%), ductal histology (86.3%), and poor differentiation (84.6%). Most patients received chemotherapy (84.8%), breast surgery (lumpectomy 45.5%, mastectomy 48.6%), and axillary surgery (SLNB 60.4%, ALND 32.0%), whereas 59.2% received radiation. Treatment was most frequently obtained at Comprehensive Community Cancer Programs (40.8%). Median survival among patients with TNBC was 58 months (25th percentile 50, 75th percentile 68). Among the 81% of patients who received chemotherapy and breast surgery, 9.7% had rapid relapse. Among the 19% of patients who did not receive these treatments, 34.7% experienced rapid relapse. All independent variables were associated with rapid relapse and receipt of treatment in bivariate analyses (chemotherapy and breast surgery; Table 2).

**Table 1:** Sociodemographic and clinical characteristics of TNBC patients in NCDB (n = 47,048)

Variable	N (%)
Rapid relapse	6,817 (14.5)
No. months survived, median (IQR)	58 (18)
Treatment (chemotherapy and breast surgery)	38,102 (81.0)
Rapid relapse with treatment	3,709 (9.7)
Did not receive both chemotherapy and breast surgery	8,946 (19.0)
Rapid relapse without treatment	3,108 (34.7)
Age group (years)	
<45	4,630 (9.8)
45–54	13,338 (28.4)
55–64	13,076 (27.8)
65–74	9,120 (19.4)
75+ years	6,884 (14.6)
Race	
American Indian or Alaska Native	143 (0.3)
Asian	1,519 (3.2)
Black	11,319 (24.1)
Hawaiian or Pacific islander	36 (0.1)
Other	409 (0.9)
White	33,622 (71.5)
Hispanic origin	3,038 (6.5)
Location of residence	
Metropolitan	41,099 (87.4)
Urban	5,281 (11.2)
Rural	668 (1.4)
Insurance	
Private/managed care	24,349 (51.8)
Medicaid	4,803 (10.2)
Medicare	15,966 (33.9)
Other government	530 (1.1)
Uninsured	1,400 (3.0)
Distance between patient’s residence and treatment facility (miles)	
<4.4	12,047 (25.6)
4.4–9	11,971 (25.4)
9–19.1	12,060 (25.6)
>19.1	10,970 (23.3)
Percentage of the population with no high school degree (area level)	
≥17.6%	10,864 (23.1)
10.9–17.5%	12,405 (26.4)
6.3–10.8%	12,687 (27.0)
<6.3%	11,092 (23.6)
Median income (area level)	
<$40,227	9,738 (20.7)
$40,227–$50,353	9,967 (21.2)
$ 50,354–$63,332	10,778 (22.9)
>$63,332	16,565 (35.2)
AJCC clinical stage	
Stage I	261 (0.6)
Stage II	38,057 (80.9)
Stage III	8,730 (18.6)
Tumor histology	
Ductal	40,582 (86.3)
Lobular	2,456 (5.2)
Others	4,010 (8.5)
Tumor grade	
Well differentiated	573 (1.2)
Moderately differentiated	6,419 (13.6)
Poorly differentiated	39,786 (84.6)
Undifferentiated	270 (0.6)
Charlson-Deyo Comorbidity Index	
0	37,936 (80.6)
1	6,778 (14.4)
≥2	2,334 (5.0)
Chemotherapy	39,882 (84.8)
Radiation	27,841 (59.2)
Breast surgery	
No surgery	2,805 (6.0)
Lumpectomy	21,401 (45.5)
Mastectomy	22,842 (48.6)
Axillary surgery	
No axillary surgery	3,568 (7.6)
Sentinel lymph node biopsy	28,435 (60.4)
Axillary lymph node dissection	15,045 (32.0)
Facility type	
Community cancer program	3,318 (7.1)
Comprehensive community cancer program	19,188 (40.8)
Academic/Research program	15,092 (32.1)
Integrated network cancer program	9,450 (20.1)

**Table 2:** Sociodemographic and clinical characteristics of rrTNBC patients and those who received treatment

Variable	Non-rrTNBC (n = 40,231); N (%)	rrTNBC (n = 6,817); N (%)	*p*	Received treatment (n=38,102); N(%)	Did not receive treatment (n=8,946); N(%)	*P*
Age group (years)			<0.001			<0.001
<45	4,172 (10.4)	458 (6.7)		4,315 (11.3)	315 (3.5)	
45–54	11,994 (29.8)	1,344 (19.7)		12,213 (32.1)	1,125 (12.6)	
55–64	11,606 (28.9)	1,470 (21.6)		11,666 (30.6)	1,410 (15.8)	
65–74	7,763 (19.3)	1,357 (19.9)		7,431 (19.5)	1,689 (18.9)	
75+	4,696 (11.7)	2,188 (32.1)		2,477 (6.5)	4,407 (49.3)	
Race			<0.001			0.001**
American Indian or Alaska Native	121 (0.3)	22 (0.3)		118 (0.3)	25 (0.3)	
Asian	1,360 (3.4)	159 (2.3)		1,257 (3.3)	262 (2.9)	
Black	9,568 (23.8)	1,751 (25.7)		9,294 (24.4)	2,025 (22.6)	
Hawaiian or Pacific islander	30 (0.1)	6 (0.1)		30 (0.1)	6 (0.1)	
Other	366 (0.9)	43 (0.6)		342 (0.9)	67 (0.8)	
White	28,786 (71.6)	4,836 (70.9)		27,061 (71.0)	6,561 (73.3)	
Hispanic origin						
Yes	2,707 (6.7)	331 (4.9)	<0.001	2,578 (6.8)	460 (5.1)	<0.001
Location of residence			<0.001			0.055*
Metropolitan	35,257 (87.6)	5,842 (85.7)		33,220 (87.2)	7,879 (88.1)	
Urban	4,424 (11.0)	857 (12.6)		4,326 (11.4)	955 (10.7)	
Rural	550 (1.4)	118 (1.7)		556 (1.5)	112 (1.3)	
Insurance			<0.001			<0.001
Private/Managed Care	21,946 (54.6)	2,403 (35.3)		22,064 (57.9)	2,285 (25.5)	
Medicaid	4,125 (10.3)	678 (10.0)		4,118 (10.8)	685 (7.7)	
Medicare	12,499 (31.1)	3,467 (50.9)		10,252 (26.9)	5,714 (63.9)	
Other Government	471 (1.2)	59 (0.9)		461 (1.2)	69 (0.8)	
Uninsured	1,190 (3.0)	210 (3.1)		1,207 (3.2)	193 (2.2)	
Distance between patient’s residence and treatment facility (miles)			<0.001			<0.001
<4.4	10,116 (25.1)	1,931 (28.3)		9,299 (24.4)	2,748 (30.7)	
4.4–9.0	10,244 (25.5)	1,727 (25.3)		9,599 (25.2)	2,372 (26.5)	
9.0–19.1	10,445 (26.0)	1,615 (23.7)		9,977 (26.2)	2,083 (23.3)	
>19.1	9,426 (23.4)	1,544 (22.7)		9,227 (24.2)	1,743 (19.5)	
Percentage of the population with no high school degree (area level)			<0.001			0.141
≥17.6%	9,217 (22.9)	1,647 (24.2)		8,755 (23.0)	2,109 (23.6)	
10.9–17.5%	10,475 (26.0)	1,930 (28.3)		9,993 (26.2)	2,412 (27.0)	
6.3–10.8%	10,867 (27.0)	1,820 (26.7)		10,309 (27.1)	2,378 (26.6)	
<6.3%	9,672 (24.0)	1,420 (20.8)		9,045 (23.7)	2,047 (22.9)	
Median income (area level)			<0.001			<0.001
<$40,227	8,096 (20.1)	1,642 (24.1)		7,808 (20.5)	1,930 (21.6)	
$40,227–$50,353	8,433 (21.0)	1,534 (22.5)		7,945 (20.9)	2,022 (22.6)	
$50,354–$63,332	9,243 (23.0)	1,535 (22.5)		8,744 (23.0)	2,034 (22.7)	
>$63,332	14,459 (35.9)	2,106 (30.9)		13,605 (35.7)	2,960 (33.1)	
AJCC clinical stage			<0.001			<0.001
Stage I	243 (0.6)	18 (0.3)		192 (0.5)	69 (0.8)	
Stage II	33,757 (83.9)	4,300 (63.1)		31,013 (81.4)	7,044 (78.7)	
Stage III	6,231 (15.5)	2,499 (36.7)		6,897 (18.1)	1,833 (20.5)	
Tumor histology			<0.001			<0.001
Ductal	34,925 (86.8)	5,657 (83.0)		33,404 (87.7)	7,178 (80.2)	
Lobular	2,055 (5.1)	401 (5.9)		1,864 (4.9)	592 (6.6)	
Other	3,251 (8.1)	759 (11.1)		2,834 (7.4)	1,176 (13.2)	
Tumor grade			0.001***			<0.001
Well differentiated	515 (1.3)	58 (0.9)		325 (0.9)	248 (2.8)	
Moderately differentiated	5,547 (13.8)	872 (12.8)		4,818 (12.7)	1,601 (17.9)	
Poorly differentiated	33,947 (84.4)	5,839 (85.7)		32,751 (86.0)	7,035 (78.6)	
Undifferentiated	222 (0.6)	48 (0.7)		208 (0.6)	62 (0.7)	
Charlson-Deyo comorbidity Index			<0.001			<0.001
0	33,041 (82.1)	4,895 (71.8)		31,383 (82.4)	6,553 (73.3)	
1	5,541 (13.8)	1,237 (18.2)		5,239 (13.8)	1,539 (17.2)	
2	1,649 (4.1)	685 (10.1)		1,480 (3.9)	854 (9.6)	
Facility type			0.005**			<0.001
Community cancer program	2,775 (6.9)	543 (8.0)		2,572 (6.8)	746 (8.3)	
Comprehensive community cancer program	16,382 (40.7)	2,806 (41.2)		15,443 (40.5)	3,745 (41.9)	
Academic/research program	12,981 (32.3)	2,111 (31.0)		12,420 (32.6)	2,672 (29.9)	
Integrated network cancer program	8,093 (20.1)	1,357 (19.9)		7,667 (20.1)	1,783 (19.9)	
Axillary surgery						
No axillary surgery	2,228 (5.5)	1,340 (19.7)	<0.001	1,032 (2.7)	2,536 (28.4)	<0.001
Sentinel lymph node biopsy	25,786 (64.1)	2,649 (38.9)		24,290 (63.8)	4,145 (46.3)	
Axillary lymph node dissection	12,217 (30.4)	2,828 (41.5)		12,780 (33.5)	2,265 (25.3)	
Radiation therapy	25,082 (62.3)	2,759 (40.5)	<0.001	25,532 (67.0)		<0.001

^**^p < 0.05; ***p ≤ 0.001

### Rapid Relapse in All TNBC Patients

With all else being constant, the probability of rapid relapse (Table 3) was higher among those 65 years and older (65–74 years by 2.0%, *p* = 0.009; ≥75 years by 8.3%, *p* < 0.001) compared with those <45 years of age (baseline risk is 14.5%; Table 3). Asian (*p* < 0.001) or Hispanic patients (*p* < 0.001) had 3.1% higher likelihood of rapid relapse compared with White and non-Hispanic patients. Patients with TNBC living in less dense neighborhoods (e.g., urban or rural) had as much as 3% higher probability of rapid relapse compared with those living in metropolitan areas. Compared with private/managed care insurance, those on Medicare and Medicaid 1.6% (*p* = 0.007) and 2% (*p* < 0.001) higher likelihood of rapid relapse, respectively. The risk of rrTNBC was highest among those without insurance with an almost 3% greater likelihood of mortality in ≤24 months (*p* = 0.005). Furthermore, patients living in neighborhoods with higher income had lower risk of developing rapid relapse (*p* < 0.001). Area-level education, facility type, and distance from treatment facility were not associated with rapid relapse (*p* > 0.05).

**Table 3:** Average marginal effects from multivariable logistic models for rapid relapse versus no rapid relapse among all patients (n = 47,048)

Variable	Average marginal effects for rapid relapse and 95% CI	
	All patients (n = 47,048)	*p*
Age group (Reference: <45 years)		
45–54	− 0.1 (− 1.2, 1.0)	0.798
55–64	0.8 (− 0.3, 1.9)	0.169
65–74	1.9 (0.5, 3.3)	0.009**
75+	8.0 (6.3, 9.7)	<0.001
Race (Reference: White race)		
American Indian or Alaska Native	3.0 (− 3.1, 9.1)	0.342
Asian	− 3.1 (− 4.6, − 1.7)	<0.001
Black	0.1 (− 0.7, 0.9)	0.852
Hawaiian and other Pacific Islander	4.7 (− 7.4, 16.8)	0.446
Other	− 2.4 (− 5.8, 0.9)	0.148
Hispanic origin	− 3.1 (− 4.5, − 1.8)	<0.001
Patient residence (Reference: metropolitan)		
Urban	1.3 (0.2, 2.5)	0.022**
Rural	3.0 (0.1, 5.8)	0.044**
Insurance (Reference: private/managed care)		
Medicaid	1.6 (0.4, 2.8)	0.007**
Medicare	2.0 (0.9, 3.0)	<0.001
Other government insurance	− 0.5 (− 3.4, 2.4)	0.754
Uninsured	2.7 (0.8, 4.5)	0.005**
Percentage of the population with no high school education (Reference: <6.3%)		
≥17.6%	− 0.4 (− 1.7, 0.8)	0.514
10.9–17.5%	0.5 (− 0.6, 1.7)	0.367
6.3–10.8%	0.8 (− 0.2, 1.7)	0.107
Area-level income (Reference: <$40,227)		
$40,227–$50,353	− 1.6 (− 2.6, − 0.5)	0.003**
$50,354–$63,332	− 1.7 (− 2.8, − 0.6)	0.003**
≥$63,333	− 2.3 (− 3.6, − 1.0)	<0.001
Distance between residence and treatment facility (<4.4 miles)		
4.4–9	− 0.1 (− 0.9, 0.8)	0.876
9.1–19.1	0.0 (− 0.9, 0.8)	0.936
>19.1	0.0 (− 1.0, 0.9)	0.933
Type of facility (Reference: academic center)		
Community cancer program	0.6 (− 0.8, 1.9)	0.401
Comprehensive community cancer program	0.3 (− 0.5, 1.1)	0.511
Integrated network cancer program	0.3 (− 0.7, 1.3)	0.590
AJCC stage (Reference: IB)		
IIA/IIB	3.2 (− 0.4, 6.7)	0.084*
IIIA/IIIB/IIIC	15.5 (11.8, 19.2)	<0.001
Tumor grade (Reference: well differentiated)		
Moderately differentiated	4.4 (2.6, 6.3)	<0.001
Poorly differentiated	7.6 (5.9, 9.4)	<0.001
Undifferentiated	8.6 (4.8, 12.4)	<0.001
Tumor histology (Reference: ductal)		
Lobular	0.7 (− 0.7, 2.2)	0.306
Others	2.3 (1.2, 3.3)	<0.001
Charlson-Deyo Comorbidity Index (Reference: 0)		
1	3.1 (2.2, 3.9)	<0.001
≥2	8.2 (6.8, 9.7)	<0.001
Radiotherapy	− 3.8 (− 4.5, − 3.1)	<0.001
Chemotherapy	− 7.9 (− 8.7, − 7.1)	<0.001
Breast surgery (Reference: no surgery)		
Lumpectomy	− 19.2 (− 21.4, − 17.1)	<0.001
Mastectomy	− 16.0 (− 18.0, − 14.0)	<0.001
Axillary surgery (no axillary surgery)		
Sentinel lymph node biopsy	− 6.2 (− 7.4, − 4.9)	<0.001
Axillary lymph node dissection	− 0.4 (− 1.7, 0.9)	0.534

^*^p < 0.10; **p < 0.05; ***p ≤ 0001

Marginal effect indicates the incremental change in the probability of rapid relapse associated with the presence or absence of a risk factor, holding all other covariates constant, e.g., patients who received chemotherapy had 7.9% (absolute difference from baseline probability: 14.5%) lower probability of rapid relapse holding all other covariates constant. Similarly, compared with those with stage IB TNBC, those with Stage III cancer had 15.5 percentage (absolute difference from baseline probability: 14.5%) higher probability of rapid relapse.

All clinical factors were associated with risk of rapid relapse. Specifically, more advanced stage of disease (stage III) had a 15.5% greater likelihood (*p* < 0.001) and poorer tumor differentiation had a successively greater likelihood of rrTNBC (*p* < 0.001). Similarly, the likelihood of rapid relapse incrementally increased with CCI (*p* < 0.001). Likelihood of rrTNBC decreased with radiation therapy (−3.8%, *p* < 0.001), chemotherapy (–7.9%, *p* < 0.001), breast surgery (lumpectomy –19.2%, *p* < 0.001; mastectomy –16%, *p* < 0.001), and SLNB (–6.2%, *p* < 0.001).

### Receipt of Treatment and Comparison of Rapid Relapse by Treatment

With all else being constant, sociodemographic factors related to receipt of both chemotherapy and breast surgery were age, race, insurance, and distance between residence and treatment facility (Table 4). The probability of receiving both chemotherapy and breast surgery decreased successively as patient age increased; patients >75 years old had a 41.3% lower probability of receiving both treatments (*p* < 0.001). Black patients with TNBC had a 1.4% lower likelihood of receiving both treatments (*p* = 0.001). Similarly, patients with Medicaid, Medicare, or no insurance were less likely to receive treatment (*p* < 0.05). Patients living further from their treatment facility were more likely to receive treatment (*p* < 0.05). Residence type, area-level education or income, and facility type had no association with treatment receipt. Patients with more advanced stages and poorer tumor differentiation had higher probabilities of receiving treatment (*p* < 0.001). However, patients with CCI ≥ 2 had 4.9% lower likelihood of receiving both treatments (*p* < 0.001).

**Table 4:** Factors associated with receiving chemotherapy and breast surgery in all TNBC patients (n = 47,048)

Variable	Average marginal effects (95% CI)	*p*
Age group (Reference: <45 years)		
45–54	− 1.8 (− 2.7, -0.9)	<0.001
55–64	− 3.7 (− 4.6, − 2.7)	<0.001
65–74	− 8.0 (− 9.3, − 6.7)	<0.001
75+	− 41.3 (− 43.5, − 39.2)	<0.001
Race (Reference: White race)		
American Indian or Alaska Native	− 4.3 (− 10.2, 1.7)	0.164
Asian	− 0.4 (− 2.2, 1.4)	0.663
Black	− 1.4 (− 2.2, − 0.6)	0.001***
Hawaiian and other Pacific Islander	− 2.6 (− 12.1, 7.0)	0.597
Other	− 2.6 (− 5.8, 0.7)	0.118
Hispanic Origin	0.8 (− 0.6, 2.2)	0.268
Patient residence (Reference: metropolitan)		
Urban	0.9 (− 0.2, 2.1)	0.098*
Rural	− 0.1 (− 2.7, 2.5)	0.946
Insurance (Reference: private/managed care)		
Medicaid	− 4.1 (− 5.2, − 3.1)	<0.001
Medicare	− 2.6 (− 3.5, − 1.6)	<0.001
Other government insurance	− 2.2 (− 4.9, 0.6)	0.125
Uninsured	− 3.4 (− 5.3, − 1.5)	0.001***
Percentage of the population with no high school education (Reference: <6.3%)		
≥17.6%	− 0.4 (− 1.6, 0.8)	0.532
10.9–17.5%	− 0.3 (− 1.4, 0.7)	0.550
6.3–10.8%	0.2 (− 0.6, 1.1)	0.589
Area-level income (Reference: <$40,227)		
$40,227–$50,353	− 0.3 (− 1.3, 0.6)	0.479
$50,354–$63,332	0.0 (− 1.1, 1.0)	0.931
≥$63,333	0.3 (− 0.9, 1.6)	0.596
Distance between residence and treatment facility (<4.4 miles)		
4.4–9	0.5 (− 0.3, 1.3)	0.236
9.1–19.1	1.1 (0.3, 1.9)	0.008**
>19.1	1.4 (0.5, 2.4)	0.003**
Type of facility (Reference: academic center)		
Community cancer program	− 0.1 (− 1.4, 1.2)	0.896
Comprehensive community cancer program	0.5 (− 0.4, 1.4)	0.321
Integrated network cancer program	0.8 (− 0.3, 1.8)	0.142
AJCC stage (Reference: IB)		
IIA/IIB	8.7 (4.5, 13.0)	<0.001
IIIA/IIIB/IIIC	7.2 (2.9, 11.5)	0.001***
Tumor grade (Reference: well differentiated)		
Moderately differentiated	11.9 (8.3, 15.4)	<0.001
Poorly differentiated	14.2 (10.7, 17.8)	<0.001
Undifferentiated	11.6 (6.2, 16.9)	<0.001
Tumor histology (Reference: ductal)		
Lobular	− 0.9 (− 2.1, 0.3)	0.160
Others	− 4.4 (− 5.5, − 3.3)	<0.001
Charlson-Deyo Comorbidity Index (Reference: 0)		
1	− 0.1 (− 0.9, 0.7)	0.858
≥2	− 4.9 (− 6.3, − 3.6)	<0.001
Radiotherapy	14.9 (14.2, 15.6)	<0.001
Axillary surgery (no axillary surgery)		
Sentinel lymph node biopsy	33.0 (31.2, 34.9)	<0.001
Axillary lymph node dissection	32.7 (30.8, 34.6)	<0.001

^*^p < 0.10; **p < 0.05; ***p ≤ 0001

Marginal effect indicates the incremental change in the probability of rapid relapse associated with the presence or absence of a risk factor, holding all other covariates constant

For example, patients who had CCI ≥2 had 4.9 percentage points lower probability of receiving both treatments holding all other covariates constant. Similarly, compared to those with stage IB TNBC, those with Stage III cancer had 7.2 percentage points higher probability of receiving treatment

### Comparison of Rapid Relapse by Treatment Receipt

With all else being constant, among patients who received both chemotherapy and breast surgery, patients older than 75 years had a 6% higher probability of rapid relapse (*p* < 0.001; Table 5). However, the probability of rapid relapse increased to 16.6%, a nearly threefold higher probability, among the same age group when no treatment was received. Similarly, patients with Medicaid and Medicare insurance each had a nearly threefold higher probability of rrTNBC between those who received and did not receive treatment. Conversely, Black (no treatment −2.8% *p* < 0.05 vs. treatment 0.5% *p* > 0.05), Asian (no treatment −7.5% vs. treatment −2.0%, *p* < 0.05 for both), or Hispanic (no treatment −5.4% vs. treatment −2.7%, *p* < 0.05 for both) patients had significantly lower likelihoods of developing rrTNBC. Patients who lived in neighborhoods with higher incomes similarly had a more than threefold lower probability of developing rrTNBC when no treatment was received (no treatment −5.2%, treatment −1.7%, *p* < 0.05 for both).

**Table 5:** Average marginal effects for patients who received both chemotherapy and surgery for TNBC (n = 38,102), and those who did not receive treatment (n = 8,946)

Variable	Average marginal effects for rapid relapse, average marginal effects (95% CI)			
	Patients who did receive treatment, percent (n = 38,102)	*p*	Patients who did not receive treatment (n = 8,946)	*p*
Age group (Reference: <45 years)				
45–54	− 0.4 (− 1.4, 0.6)	0.392	3.0 (− 1.6, 7.6)	0.201
55–64	0.1 (− 0.9, 1.2)	0.826	6.1 (1.3, 10.9)	0.012**
65–74	1.1 (− 0.2, 2.5)	0.107	7.3 (2.2, 12.4)	0.005**
75+	6.0 (4.1, 7.9)	<0.001	16.6 (11.5, 21.8)	<0.001
Race (Reference: White race)				
American Indian or Alaska Native	3.3 (− 2.7, 9.3)	0.275	− 0.4 (− 18.0, 17.2)	0.967
Asian	− 2.0 (− 3.4, − 0.6)	0.005**	− 7.5 (− 12.3, − 2.7)	0.002**
Black	0.5 (− 0.2, 1.3)	0.161	− 2.8 (− 5.3, − 0.2)	0.035**
Hawaiian and other Pacific Islander	8.9 (− 4.9, 22.7)	0.204	− 14.4 (− 40.4, 11.6)	0.278
Other	− 1.1 (− 4.3, 2.1)	0.502	− 7.6 (− 18.4, 3.2)	0.166
Hispanic origin	− 2.7 (− 4.1, − 1.3)	<0.001	− 5.4 (− 9.9, − 0.9)	0.020**
Patient residence (Reference: metropolitan)				
Urban	0.8 (− 0.2, 1.9)	0.129	3.2 (− 0.3, 6.8)	0.076*
Rural	2.7 (− 0.3, 5.8)	0.081*	3.6 (− 5.8, 13.0)	0.451
Insurance (Reference: private/managed care)				
Medicaid	1.2 (0.2, 2.3)	0.022**	3.6 (− 0.3, 7.5)	0.068*
Medicare	1.6 (0.5, 2.7)	0.004**	3.6 (0.6, 6.7)	0.020**
Other Government Insurance	− 0.5 (− 3.1, 2.0)	0.687	− 0.8 (− 11.6, 10.0)	0.882
Uninsured	2.3 (0.5, 4.1)	0.010**	4.5 (− 1.6, 10.6)	0.149
Percentage of the population with no high school education (Reference: <6.3%)				
≥17.6%	0.0 (− 1.1, 1.2)	0.935	− 2.5 (− 6.3, 1.4)	0.212
10.9–17.5%	0.9 (− 0.2, 2.0)	0.124	− 0.9 (− 4.2, 2.5)	0.606
6.3–10.8%	1.1 (0.2, 2.0)	0.014**	− 1.0 (− 3.8, 1.8)	0.486
Area-level income (Reference: <$40,227)				
$40,227–$50,353	− 1.3 (− 2.3, − 0.3)	0.014**	− 3.0 (− 6.0, 0.0)	0.050**
$50,354–$63,332	− 1.3 (− 2.5, − 0.2)	0.023**	− 3.6 (− 7.0, − 0.2)	0.036**
≥$63,333	− 1.7 (− 3.0, − 0.5)	0.006**	− 5.2 (− 8.9, − 1.4)	0.008**
Distance between residence and treatment facility (<4.4 miles)				
4.4–9 miles	− 0.2 (− 1.0, 0.7)	0.676	0.4 (− 2.0, 2.8)	0.750
9.1–19.1 miles	0.2 (− 0.7, 1.1)	0.681	− 0.6 (− 3.2, 2.0)	0.653
>19.1 miles	0.7 (− 0.3, 1.6)	0.166	− 2.9 (− 6.0, 0.2)	0.064*
Type of facility (Reference: Academic Center)				
Community Cancer Program	0.5 (− 0.8, 1.8)	0.428	0.7 (− 3.3, 4.8)	0.727
Comprehensive Community Cancer Program	0.3 (− 0.5, 1.0)	0.450	0.7 (− 1.9, 3.3)	0.599
Integrated Network Cancer Program	0.2 (− 0.7, 1.0)	0.721	1.1 (− 1.8, 4.1)	0.454
AJCC Stage (Reference: IB)				
IIA/IIB	0.5 (− 3.3, 4.4)	0.782	14.0 (3.6, 24.4)	0.008**
IIIA/IIIB/IIIC	11.5 (7.5, 15.5)	<0.001	36.4 (25.8, 47.0)	<0.001
Tumor grade (Reference: Well differentiated)				
Moderately differentiated	2.9 (0.5, 5.3)	0.020**	9.2 (4.1, 14.3)	<0.001
Poorly differentiated	4.8 (2.5, 7.2)	<0.001	17.0 (12.1, 22.0)	<0.001
Undifferentiated	5.4 (1.1, 9.7)	0.015**	20.2 (8.9, 31.5)	<0.001
Tumor histology (Reference: ductal)				
Lobular	1.5 (0.0, 3.0)	0.044**	− 1.2 (− 5.0, 2.7)	0.558
Other	2.7 (1.4, 3.9)	<0.001	2.1 (− 0.7, 4.9)	0.134
Charlson-Deyo Comorbidity Index (Reference: 0)				
1	1.4 (0.5, 2.2)	0.003**	9.9 (7.4, 12.5)	<0.001
≥2	5.9 (4.1, 7.7)	<0.001	16.6 (13.3, 20.0)	<0.001
Radiotherapy	− 4.5 (− 5.1, − 3.9)	<0.001	− 7.2 (− 9.6, − 4.8)	<0.001
Axillary surgery (no axillary surgery)				
Sentinel lymph node biopsy	− 2.6 (− 4.4, − 0.9)	0.003**	− 11.2 (− 13.8, − 8.7)	<0.001
Axillary lymph node dissection	3.0 (1.2, 4.8)	0.001***	− 3.4 (− 6.2, − 0.6)	0.019**
Chemotherapy	–	–	− 12.0 (− 15.2, − 8.8)	<0.001
Breast surgery (Reference: no surgery)				
Lumpectomy	–	–	− 25.4 (− 29.0, − 21.7)	<0.001
Mastectomy	–	–	− 22.5 (− 25.9, − 19.0)	<0.001

**p < 0.05; ***p < 0.001; *p < 0.100

With all else being constant, patients with more advanced disease (Stage III vs. Stage IB) who did not receive treatment had a nearly four times higher likelihood of developing rrTNBC (no treatment 36.4% vs. treatment 11.5%, *p* < 0.001). Moreover, among those with poorer tumor differentiation, those who did not receive treatment had a 3.7 times higher likelihood of developing rrTNBC (no treatment 17.0% vs. 4.8%, *p* < 0.001). Patients with higher CCI scores similarly had a nearly threefold higher likelihood of rapid relapse when no treatment was received (*p* < 0.001).

## Discussion

Our study sought to better understand risk factors that contribute to the development of rapid relapse in patients with triple-negative breast cancer. Among patients with TNBC in the NCDB, 14.5% experienced rapid relapse, many of whom were older, lived in rural areas, were publicly insured, and had more comorbidities. Patients with similar clinical and demographic factors were less likely to receive chemotherapy and breast surgery. Most importantly, our study found that patients who did not receive these treatments were nearly four times as likely to develop rapid relapse (34.7% vs. 9.7%). Furthermore, patients who did not receive both chemotherapy and breast surgery were more likely to be 75 years or older, have Stage III cancer, or have multiple comorbidities. These patients also were three times more likely to experience rapid relapse compared with those younger than 45 years, having Stage IB cancer, or no comorbidities. Our study suggests that receiving both chemotherapy and breast surgery may be protective against the development of rapid relapse.

In comparison with extant literature, the prevalence of rapid relapse in our study was almost twice that reported by the SEER (8%) study, while being slightly less than in the NCCN (16.9%) study.^8,9^ These differences in the three studies might be the result of how rrTNBC was operationalized to leverage strengths of the underlying data, as well as population differences across the three studies. While our study defined rrTNBC using all-cause mortality, the SEER study was able to use disease-specific mortality captured in that data.^8^ Conversely, the NCCN study included distant metastatic recurrence at ≤24 months in their definition.^9^

The differences in the samples might explain relationships observed across the three studies. For example, older age was related to higher likelihood of rrTNBC in our study, but no such association was seen in SEER or the NCCN.^8,9^ Both studies based on SEER and the NCCN represent a relatively younger sample with few patients older than 70 years. In contrast, more than a third of our sample was 65 years and older. Additionally, although Black race was associated with greater rapid relapse in SEER,^8^ no such relationship was observed in our analysis. Moreover, we found that Asian patients were less likely to develop rapid relapse, whereas no studies have previously evaluated the prevalence of rrTNBC in the Asian community. These differences from extant literature may be attributed to sociodemographic characteristics of racial minorities in our study regarding age, insurance coverage, and access to care. We also found that patients with more comorbidities were more likely to develop rapid relapse, whereas comorbidity was not an independent predictor of rrTNBC in the NCCN.^9^

The higher likelihood of rapid relapse among patients with public or no insurance is consistent with previous studies.^8,9^ Patients with public or no insurance tend to forgo care, including preventative services, because of prohibitive costs or lack of access to health care, which contributes to their presentation at a more advanced stage of disease at diagnosis.^22–25^ This is supported by the finding that rural Americans had the greatest likelihood for rapid relapse. Rural Americans face inequities, such as lack of insurance, socioeconomic deprivation, and physician shortages,^26^ which may result in worse health care than their urban counterparts as well as less likely to use preventive care.^27,28^ In the same vein, we found that patients who lived in areas with higher area-level income, and thereby likely greater access to resources, had a lower likelihood of developing rapid relapse. Collectively, these findings suggest that neighborhood contextual factors may play a role in the likelihood of rapid relapse. Patients with more advanced tumor stage and poorer tumor differentiation had a higher probability of rapid relapse, which is consistent with findings from SEER and the NCCN.^8,9^ We also found that receipt of all three treatments (breast surgery, chemotherapy, and radiation therapy) was associated with lower risk of rrTNBC. In the SEER study, breast surgery and radiation therapy were independently associated with rrTNBC. Conversely, the NCCN did not examine the influence of breast surgery, and radiation therapy was not independently associated with rrTNBC, making this study one of the first to evaluate the risk of rrTNBC and all three treatment modalities concomitantly.

An important contribution of our study is examining the relationship between receipt of both breast surgery and chemotherapy by patients with TNBC and the probability of rapid relapse in relation to treatment receipt. Black patients, those with public or no insurance, or greater comorbidities were less likely to receive both breast surgery and chemotherapy together. Notably, we found that patients who lived closer to their treatment facility were less likely to receive both chemotherapy and breast surgery. While the reason for this association is unclear, previous studies have suggested that Black patients are frequently siloed into urban regions (i.e., through redlining), placing them geographically closer to hospitals in the NCDB, which also are mostly located in urban areas,^29,30^ which would further contribute to the racial differences in treatment receipt. For those patients who had public insurance or were uninsured, the likelihood of rapid relapse was two to three times higher among patients who did not receive treatment. The National Surgical Adjuvant Breast and Bowel Project’s recent findings suggest that receipt of treatment eliminated racial disparities in survival. Thus, our findings lend support for the role of SDH as contributing factors in access to care and underscore the importance of ensuring equitable access to treatment among marginalized communities.^31^

Our study found that the likelihood of receiving both chemotherapy and surgery declined as patients aged. This is consistent with evidence from extant literature, which reports lower likelihood of receiving chemotherapy among patients with TNBC who had higher comorbidity. These patients also subsequently had higher mortality. However, among older populations who did receive treatment, the likelihood of rrTNBC decreased by nearly threefold compared with those who did not. Similarly, patients with Medicare insurance and higher comorbidity were less likely to develop rapid relapse when they received treatment. Extant literature has found similar associations whereby patients with TNBC and higher comorbidity were less likely to receive both treatments and consequently had higher mortality rates.^32^ Additionally, previous research on breast cancer has shown that older patients in good health may tolerate chemotherapy as well as younger patients.^33^ While older patients may be deemed unfit to tolerate treatment, particularly given their greater comorbidities,^34–36^ more selective exclusion criteria may be necessary given the significant benefit of treatment seen in older populations.

However, our findings suggest that receiving treatment may play a less beneficial role in the likelihood of rrTNBC in minoritized populations. Although Black patients were less likely to receive treatment, those who did not receive treatment were less likely to have rapid relapse. Similarly, Asian patients and Hispanic patients who did not receive treatment were nearly four times and two times less likely to have rrTNBC than those who received treatment, respectively. Current best practices and treatment guidelines for management of TNBC are based on studies on samples that are predominantly White with low representation of Asian populations. Additionally, Asian race made for less than 5% of our sample—less than the national average. For these reasons, it is likely that our findings regarding Asian race may warrant a deeper investigation. Our findings suggest the need for future studies investigating whether current treatment protocols for TNBC are as effective amongst minority populations.

Our findings suggest that SDH may play a lesser role in rapid relapse when patients do receive guideline recommended treatment. We found that higher area-level income was associated with a probability of rapid relapse that was three times lower in patients who did not receive treatment, thereby emphasizing the role of access to treatment. These SDH-related findings may be explained by the type of care available to patients at facilities represented in the NCDB, which are required to provide care that is aligned with treatment guidelines to maintain their CoC accreditation. As such, we noted no differences in the probability of rrTNBC based on the type of facilities patients received their treatment.

Findings from our study have implications for clinical practice and research directions. First, receipt of both chemotherapy and breast surgery may protect against the development of rrTNBC. Second, although patients who were older, on public insurance, and had greater comorbidity were less likely to receive chemotherapy and breast surgery, when they did receive both treatments, they had three to four times lower chance of rapid relapse. These findings may inform patient and provider engagement strategies to fully consider the potential benefits of both treatments in older, sicker patients on public insurance. Use of shared decision-making approaches that encourage communication between care providers and patients and coordinated care by multidisciplinary teams may be helpful in this context. Third, our findings highlight the need for studying the efficacy of treatment guidelines in diverse patients. Fourth, our findings indicate that more research is needed on how increasing the availability of treatments could reduce the risk of developing rapid relapse, especially for patients with public or no insurance who receive treatment for TNBC. Lastly, further studies are needed to better evaluate the impact of neighborhood contextual factors on the development of rrTNBC.

### Limitations

Our study has the following limitations. First, we used all-cause mortality at ≤24 months as a measure of rapid relapse, because NCDB does not have information on cause-specific mortality. However, because the focus of our analysis is a particularly aggressive subtype of TNBC, it is likely that patients who died at ≤24 months did so from rrTNBC.y^6^ Our analysis controlled for demographic factors and comorbidities. Second, data in the NCDB comes from CoC accredited hospitals, which are more likely to provide guideline-concordant care. Therefore, it is likely that the observed associations may be underestimated, particularly as they apply to non-CoC accredited facilities. Future studies may explore these findings by examining real-world data, such as electronic health records that captures cause specific mortality, care-related factors from both CoC and non-CoC hospitals, as well as clinical factors including comorbidities. Third, the sample size for individuals of Asian race did not reflect the national average (0.63% vs. 7.3%), potentially impacting the reliability of the findings. Additionally, the racial distribution of patients who present to CoC accredited hospitals may not align with the true national distribution of TNBC amongst racially minoritized groups, which may influence the generalizability of our findings to these specific groups. Therefore, our findings related to the probability of rapid relapse among Asian and Black patients, who did not receive both surgery and chemotherapy, must be interpreted with caution. Fourth, the anonymity of facilities in the NCDB also limits our ability to control facility characteristics in our analysis. However, we used robust standard errors to account for heteroscedasticity arising from clustering of outcomes within the same facility. We did not assess the receipt of radiation therapy along with breast surgery and chemotherapy due to limitations in delineating the indications and timing of receipt relative to surgical management. Lastly, our analysis may be subject to selection bias as with any retrospective data analysis. However, we have attempted to minimize this by adjusting for relevant independent variables.

## Conclusions

Our study found disparities in the receipt of breast surgery and chemotherapy among older patients, of Black race, with Medicaid insurance, and greater comorbidity. Not receiving both treatments was associated with a two-to-three-fold higher probability of rrTNBC among patients ≥75 years, on Medicare, and higher comorbidity. These findings present implications for care delivery as well as future research directions.
